# Reversing 21 years of chronic paralysis via non‐invasive spinal cord neuromodulation: a case study

**DOI:** 10.1002/acn3.51051

**Published:** 2020-05-20

**Authors:** Monzurul Alam, Yan To Ling, Arnold Y. L. Wong, Hui Zhong, Victor Reggie Edgerton, Yong‐Ping Zheng

**Affiliations:** ^1^ Department of Biomedical Engineering The Hong Kong Polytechnic University Kowloon Hong Kong; ^2^ Department of Rehabilitation Sciences The Hong Kong Polytechnic University Kowloon Hong Kong; ^3^ Department of Neurobiology University of California Los Angeles CA; ^4^ Department of Integrative Biology and Physiology University of California Los Angeles CA United States

## Abstract

**Objective:**

The objective of the current study was to investigate if a non‐invasive spinal cord neuromodulation modality could restore sensorimotor functions in a patient with chronic spinal cord injury (SCI).

**Methods:**

In this study, transcutaneous electrical stimulation (tES) to the spinal cord was utilized to restore sensorimotor functions in a chronic SCI patient who sustained a traumatic C7 cervical cord injury 21 years ago. At baseline, the patient had very limited volitional movement in her right leg, and her left leg was completely paralyzed. tES parameters were optimized in eight stimulation sessions before the treatment. The therapeutic stimulation involved biphasic tES, applied to T11 and L1 spinal levels during a 1‐hour standing and walking training, 2–4 times per week for 16 weeks.

**Results:**

Our pre‐treatment tests indicated that a shorter burst duration (100 *µ*sec) was more effective than a longer burst duration of tES in improving functional movements. After 32 training sessions with tES, the patient regained significant left‐leg volitional movements (grade 0 to grade 10 according to the ISNCSCI scale). Right‐leg motor scores also increased from 17 to 21. The tES treatment also improved her pinprick sensation (from 73 to 79). Upon completion of the treatment (52 sessions), the patient’s standing ability noticeably improved. She could stabilize her knee to stand without any assistance. She could also squat while holding onto a walker.

**Interpretation:**

These promising results demonstrate beneficial effects of non‐invasive tES in regaining volitional control of plegic lower limbs in patients with chronic paralysis.

## Introduction

Millions of patients are suffering from paralysis worldwide following traumatic spinal cord injury (SCI)^1^, and an estimated 768,473 new patients are adding to this number each year.^2^ Although there is no complete recovery from paralysis yet, recent groundbreaking studies on epidural electrical stimulation (eES) of the lumbosacral spinal cord have successfully restored voluntary control of paralyzed limbs,^3^, ^4^ full weight‐bearing standing and over‐ground stepping^5^, ^6^ in chronic motor‐complete SCI patients when combined with extensive physical therapy and motor‐training. However, eES of the spinal cord requires surgical implantation of a neurostimulator and associated stimulation electrodes into the patient, which may cause complications.

Transcutaneous electrical stimulation (tES) is a non‐invasive neuromodulation method to activate neural circuits via electric current between a pair of stimulating electrodes placed onto the skin. The tES to SCI patients has recently demonstrated successful activation of motor pools of distal muscles,^7^ facilitation for standing,^8^ and inducing stepping‐like movements in a gravity‐neutral position.^9^ However, tES has not yet exhibited full restoration of voluntary movements of paralyzed limbs, independent full‐weight‐bearing standing, or bipedal stepping without any assistance in paralyzed patients. Herein we present a chronic SCI patient with lower‐extremity monoplegia, who had been wheelchair bound for the last 21 years since a traumatic cervical cord injury following a traffic accident, regained full volitional movements of her paralyzed leg, independent standing and squatting after 16 consecutive weeks (52 sessions) of weight‐bearing standing and assisted stepping training with non‐invasive spinal cord stimulation (tES).

## Methods

The study was granted approval by the Hong Kong Polytechnic University Human Subjects Ethics Sub‐committee, and was registered on the ClinicalTrials.gov (Identifier: NCT04171375).

### Study participant

The participant was a 48‐year‐old woman who suffered a C7 cervical cord injury (burst fracture) in a road traffic accident 21 years ago. Most of her upper limb functions had been retained for moderate daily activities, although her distal muscles remained affected, and the patient could not completely extend or flex her fingers. Some of her trunk controls had also been retained, allowing her to sit independently with her hands partially supporting her upper‐extremity weight. However, her lower limb functions had been significantly lost. Specifically, her right leg had very limited voluntary movements, and the left leg was completely paralyzed at the beginning of the study. Both legs demonstrated muscle spasms, particularly the left leg. Some bladder functions had been retained although the sensations were weak and altered.

### Procedures

**Figure 1:**
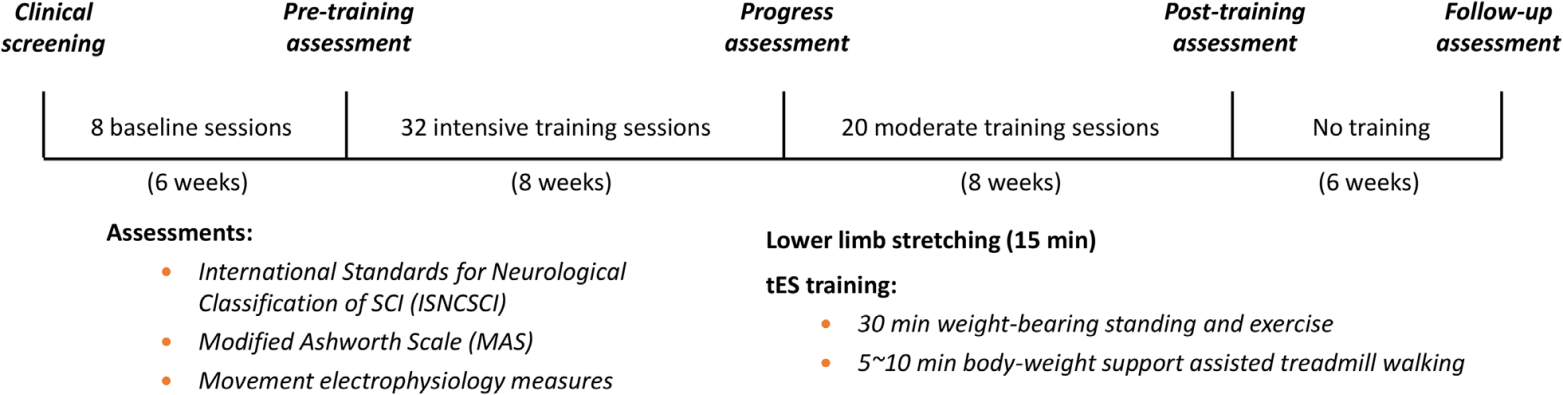
Study procedures. After passing the clinical screening, eight transcutaneous electrical stimulation (tES) sessions were conducted on the study participant to determine the optimum stimulation parameters for training, followed by pre‐training assessments (listed in the figure). tES sessions were then applied 4 times/week in conjunction with physical training. After 32 intensive training sessions, functional reassessments were conducted to determine the participant’s improvement. Upon completion of the 32 intensive training and progress assessments, the participant underwent another 20 moderate training sessions (2–3 times/week) with tES before the post‐training assessment. Furthermore, 6‐week post‐treatment follow up was conducted to assess the participant’s functional ability.

After passing the initial screening for cardiac health and bone density assessment for exercise by an independent physician, the patient was recruited into the tES study with informed consent. The outline of the study procedure is shown in Figure 1. The number of training sessions, their duration and intensity were determined based on the protocols used in previous stimulation and training studies^10^, ^11^ on similar patients. Following pre‐training assessments, the participant attended eight baseline sessions to determine the optimum tES parameters for training. Then the participant attended 2‐h training sessions at a frequency of 2–4 sessions per week for 16 consecutive weeks. Three 5‐min stretching (a total of 15 min) sessions with 2–3 min of rest in between were provided to the patient before each tES training session. Each training session was divided into two segments in the following order: (1) three 10‐min assisted standing sessions (a total of 30 min) with 3–5 min of break, and (2) three 2–3 min of 20–30% body‐weight supported assisted treadmill walking (a total of 5–10 min) with 1–2 min of rest in between. Blood pressure was measured regularly between segments. As the standing training progressed, the patient was asked to perform trunk and lower‐body exercises such as side bending while holding dumbbells and squatting. The duration of the walking exercise also progressed gradually according to the participant’s condition.

### Stimulation protocol

In this study, two specially programmed constant current stimulators (DS8R, Digitimer, UK) were used to simultaneously stimulate the participant’s T11 and L1 segments. The stimulators were triggered by a function generator (AFG1022, Tektronix, Inc.), generating a 9.4 kHz burst signal delivered at 0.5–30 Hz. Two burst configurations were tested: 1 biphasic pulse (101 *µ*sec burst duration, hereafter referred as 100 *µ*sec tES) and 10 biphasic pulses (1.06 msec burst duration, hereafter referred as 1 msec tES). The pulse durations of each phase were kept constant to 50 *µ*sec for both stimulation configurations. The tES was delivered at an intensity ranging from 20 to 120 mA by two self‐adhesive stimulation electrodes with a diameter of 3.2 cm (ValuTrode, Axelgaard Manufacturing Co. Ltd.) placed at the midline immediately below the T11 and L1 spinous processes (hereafter referred to as T11 and L1 electrodes) and two internally connected 6.0 × 9.0 cm self‐adhesive rectangular return electrodes (Guangzhou Jetta Electronic Medical Device Manufacturing Co. Ltd., China) placed on the skin over the iliac crests.

### Standing training with tES

After several iterations during the first 6 weeks of the study, optimal stimulation parameters were selected based on the participant’s comfort and the perceived ease of standing with least supports. The participant was blinded to the parameter changes, but was aware of the stimulation and gave verbal feedback to the investigator. Three 10‐min (a total of 30 min) standing training segments were provided per training session (Fig. 1). The tES parameters included 20 Hz tonic biphasic stimulation with 100 *µ*sec burst duration delivered simultaneously at the T11 level with 105 mA and at the L1 level with 100 mA currents, respectively. The stimulation parameters were kept constant throughout the entire training period. Manual supports were provided to the feet, knee, and pelvis at the early stage of training, and gradually withdrawn with time as the standing stability improved. For the last 20 training sessions, during standing training, the patient was trained to perform squats. The same stimulation parameters were adopted for the squat training.

### Treadmill walking training with tES

In this study, tES was used to assist our participant during body‐weight supported treadmill walking. Two trainers assisted the participant to place her legs to mimic a normal gait cycle on a moving treadmill belt (1.125 km/h), while one trainer emulated the pelvis rotation during walking. After several sessions during the first 6 weeks of the study, the “best” stimulation parameters were chosen by the participant using the same strategy for selecting the standing stimulation parameters. The selected tES parameters for walking were 30 Hz tonic biphasic stimulation with 100 *µ*sec burst duration delivered simultaneously at T11 with 95 mA and L1 with 90 mA currents. The patient underwent three 2–3 min (a total of 5–10 min) of walking training per training session. The stimulation parameters were kept constant throughout the training sessions.

### Testing of volitional movements with and without tES

In this study, tES was utilized to restore volitional control of the patient. Tonic biphasic stimulation with 100 *µ*sec to 1 msec burst duration was delivered simultaneously at T11 and L1 spinal segments at 20–30 Hz. Stimulation current was varied from 20–120 mA and the patient was encouraged to try to move her legs. At the early stage (first 8 baseline sessions) of the study, manual support was provided in the form of a tendon press if the patient was unable to move her leg voluntarily (see Video S1). After every 4‐week motor training, the patient was retested for voluntary lower limb movements in supine and sitting positions. At the end of the training, the patient was also tested for squatting with and without tES.

### Data acquisition and analysis

An integrated motion capture system (Vicon Nexus, Vicon Motion Systems Ltd., UK) with a floor‐mounted force plate (OR6, Advanced Mechanical Technology Inc.) and 8‐channel wireless electromyography (EMG) acquisition (BTS Telemg, BTS Engineering) was used to capture body kinematics and lower limb muscles activities. Eight pairs of Ag/AgCl button electrodes (YD30‐6, YanchengTianrun Medical Instrument Factory) were placed on the muscle bellies of the quadriceps, tibialis anterior, hamstrings, and gastrocnemius of both legs, respectively. The EMG signal was digitized at a 2 kHz sampling rate and stored in a computer for offline analysis. During sit‐to‐stand and stand‐to‐sit tasks, the participant’s load on her feet and the change in center of pressure were measured using the force plate. Videos were also taken during different tasks using a digital camera and analyzed along with the kinematic data.

EMG, force, and kinematic data were analyzed offline using customized written scripts in MATLAB (MathWorks Inc., Natick, MA). Peak‐to‐peak values of motor evoked potential (MEP) were measured and plotted against the normalized tES current intensities to determine the recruitment of muscles for each stimulation configuration (100 *µ*sec and 1 msec tES bursts). Repeated measures analysis of variance (ANOVA) was used to determine the overall difference among the scores at different time points. The significance level was set at 0.05. All statistical analyses were performed using Prism (GraphPad Software Inc., La Jolla, CA).

## Results

### Effects of tES parameters on lower‐extremity motor pools

**Figure 2:**
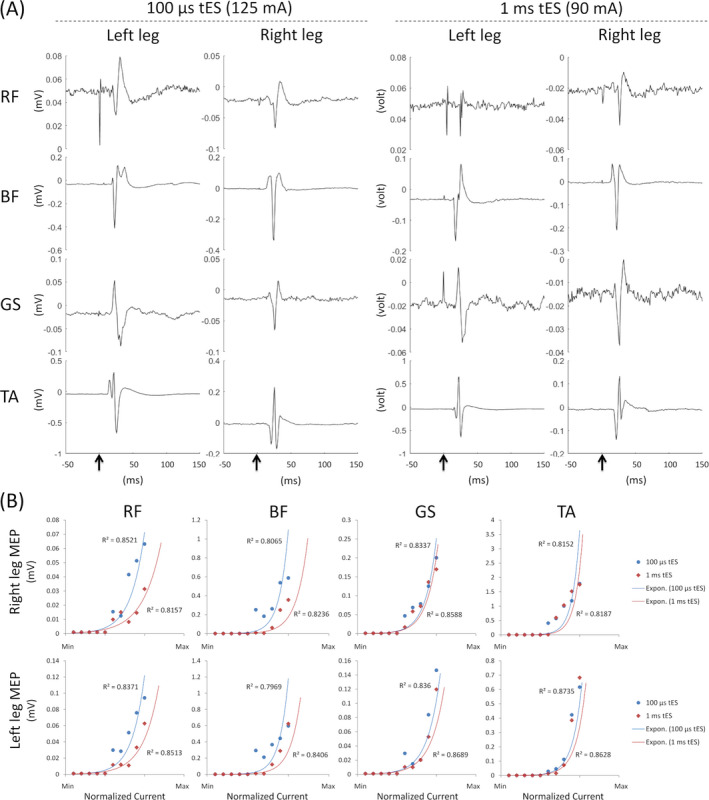
Transcutaneous electrical stimulation (tES) at T11 and L1 spinal level induced motor evoked potential (MEP) in different lower‐limb muscles every 2 sec. (A) MEP induced with 100 *µ*sec at 125 mA and with 1 msec tES at 90 mA stimulation intensities. (B) Motor recruitments were calculated from peak‐to‐peak amplitude of MEP signals at different stimulation intensities for both stimulation configurations (100 *µ*sec and 1 msec tES settings). Each *R* ^2^ value indicates the exponential fit of motor recruitments. RF, rectus femoris, BF, biceps femoris; GS, gastrocnemius; TA, tibialis anterior.

Transcutaneous electrical stimulation (tES) simultaneously delivered at T11 and L1 spinal segments evoked muscle contractions in both legs. To find the optimal tES configuration to activate the most lower‐extremity motor pools, two tES settings were tested: 100 *µ*sec burst and 1 msec burst of stimulations (see the Methods section). Figure 2A shows the raw MEP signals for 100 *µ*sec tES at 125 mA, and 1 msec tES at 90 mA constant stimulation currents. As expected, due to the longer burst duration, comparatively less tES current was required to induce an MEP for the 1 msec tES setting. The MEP signals appeared to be similar for both configurations; however, the peak‐to‐peak MEP values were more consistent for 100 *µ*sec tES as compared to 1 msec tES (Fig. 2B). The motor recruitment curves indicated by the exponential fits show little or no difference between the two tES configurations (100 *µ*sec vs. 1 msec burst durations). However, the recruitment curves for the proximal muscles (rectus femoris and biceps femoris) for the 1 msec burst setting exhibited a slower rise and thus diverged from the recruitment curves for the 100 *µ*sec burst setting. 100 *µ*sec tES thus exhibited more homogeneous recruitments of all the muscles tested.

To facilitate more muscle recruitments in our patient, we selected 100 *µ*sec tES treatment at a comfortable stimulation intensity (90–105 *µ*A) throughout the study. At the baseline sessions of tES parameter screening (see Fig. 1), we found that 100 *µ*sec tES at 30 Hz could instantaneously alter the function of the left paralyzed limb and allowed the motor volley to induce volitional movements of the paretic muscles. The movements of the left paralyzed limb could be further enhanced by a knee tendon press during the spinal cord stimulation; however, the movements were completely absent in the absence of the tES (Video S1).

### Restoration of volitional movements of the patient’s paralyzed limb

After 2–3 weeks of assisted standing and body‐weight supported treadmill walking along with the spinal cord stimulation (100 *µ*sec tES), it was observed that the patient started to move her plegic left leg although she was unaware of her movements. Therefore, we placed a large mirror in front of the patient to help her observe her own volitional movements to enhance her confidence in the training. After 20 intensive training sessions with tES, the patient started to regain some sensations during her volitional leg movements and could selectively control each leg movement even in the absence of tES (Video S2).

**Figure 3:**
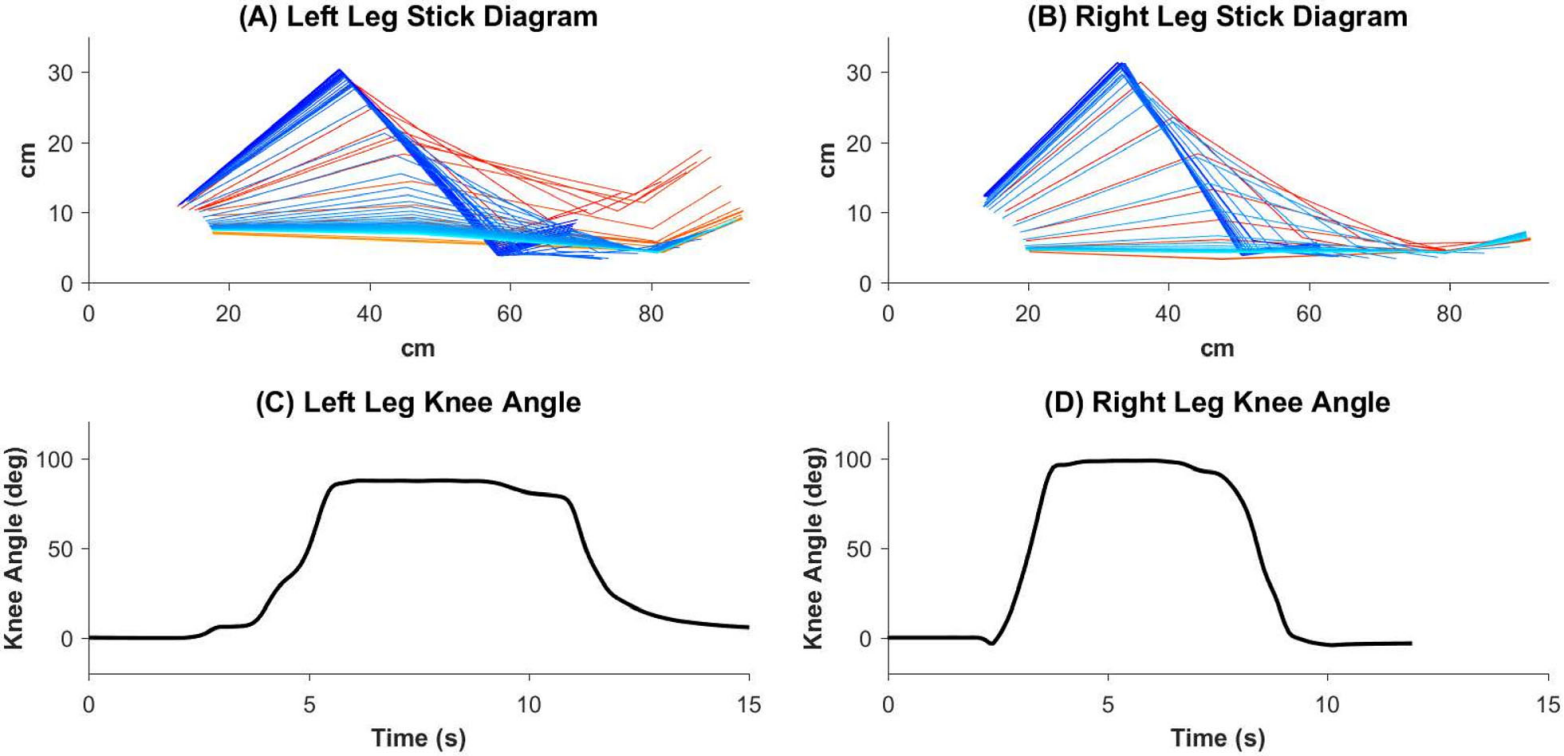
Volitional hip and knee flexion and extension movements without stimulation in the supine position after 32 sessions of tES treatment and training. (A and B) Stick diagrams in sagittal view. Motion tracking reflective markers were placed on the upper lateral 1/3 surface of the thigh, lateral femoral epicondyle, medial femoral epicondyle, medial malleolus, and the second metatarsal head. The motions of the knee moving in the cephalic and caudal directions are plotted from yellow‐to‐red, and from blue‐to‐cyan respectively. Each line plotted is separated in time by 200 msec. (C and D) Knee flexion angles calculated from the motion tracking markers, where zero degree marks angle at the initial resting state, positive slope represents knee flexion, and negative slope represents knee extension.

At the end of 32 intensive training sessions with tES, the patient was able to volitionally flex and extend both knees, for the first time, without any stimulation. Figure 3 shows the patient’s volitional movements of hip and knee flexion and extension in a supine position, after 32 sessions of training with tES treatment. The patient moved her left leg by initiating hip flexion with minimal knee flexion, resulting in foot lifting. The right leg moved faster with regard to moving from full extension to full knee flexion and back to full extension, which took 2.56 sec and 3.76 sec, respectively. In comparison, the left leg took 4 sec and 6.12 sec to achieve the same movements. The knee flexion angle of the left and right leg reached a maximum of 87° and 98°, respectively.

### Improvements of weight‐bearing standing and squatting with tES

**Figure 4:**
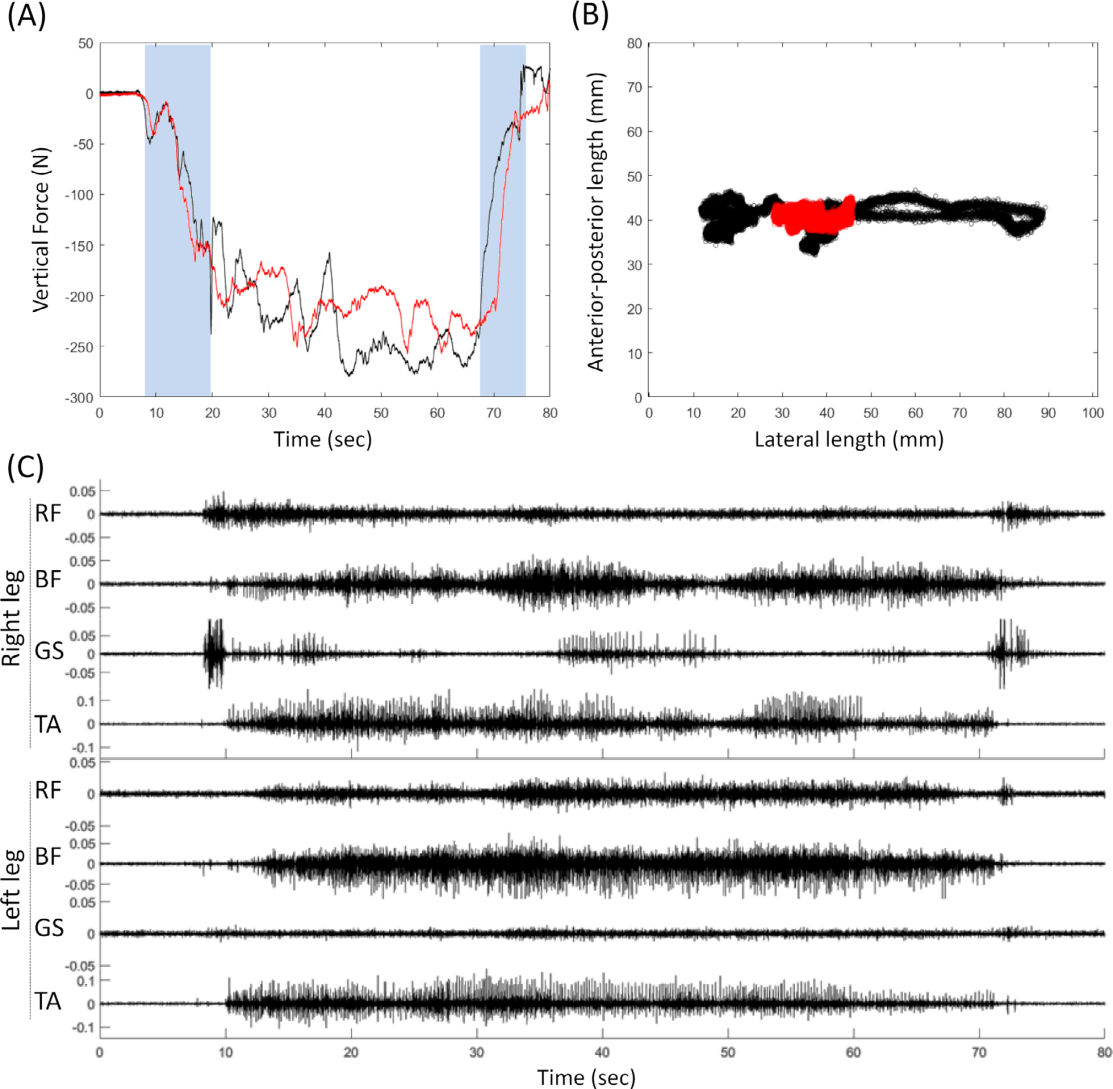
Independent sit‐to‐stand and stand‐to‐sit function with the aid of a walker after 32 treatment sessions. (A) Vertical force acted by the patient onto a floor‐embedded force plate. The shaded areas indicate sit‐to‐stand and stand‐to‐sit transitions, respectively; and (B) changes in the center of mass with (red) and without (black) tES. (C) surface electromyography amplitudes (mV) during the whole sit‐to‐stand and stand‐to‐sit task without stimulation.

Full weight‐bearing standing of the participant was facilitated by 20 Hz tES. After 32 standing training sessions with tES, the patient was able to lock both knees during standing with the aid of a walker only. Figure 4A shows the vertical force exerted onto the floor by the patient during independent sit‐to‐stand and stand‐to‐sit tasks with and without tES. Although the ground reaction force appeared to be similar with and without the spinal cord stimulation (Fig. 4A), the center of mass was found to be more centered during standing with tES as compared to standing without tES (Fig. 4B), suggesting better standing stability with tES. The surface EMG signals, however, did not exhibit good synergies and demonstrated significant co‐activations of antagonist muscles during the sit‐to‐stand and stand‐to‐sit tasks (Fig. 4C). Nonetheless, after 32 sessions of tES treatment with standing training, the patient could stand in an upright posture with minimal manual supports to her feet to prevent excessive inversion and plantar flexion of her ankles.

After 32 intensive training sessions, we further trained the patient to stand independently and to try to perform trunk and lower‐body exercises during standing with spinal cord stimulation (tES) at T11 and L1 spinal levels. The patient was able to perform trunk lateral bending with occasional involuntary flexing of the opposite knee. However, knee buckling could be self‐corrected and improved as the training progressed. After approximately 40 training sessions using 20 Hz tES, the patient was able to squat with her hands supported on the handle of a walker for the first time since her injury. Upon completion of 52 tES training sessions, the patient could perform independent short‐squats without hand support as well as deep‐squats with the support of her hands on the handle of a walker even in the absence of tES (Video S3). This dramatic improvement in her volitional function significantly boosted her self‐confidence in recovery.

### Permanent recovery from paralysis with spinal cord stimulation and training

**Figure 5:**
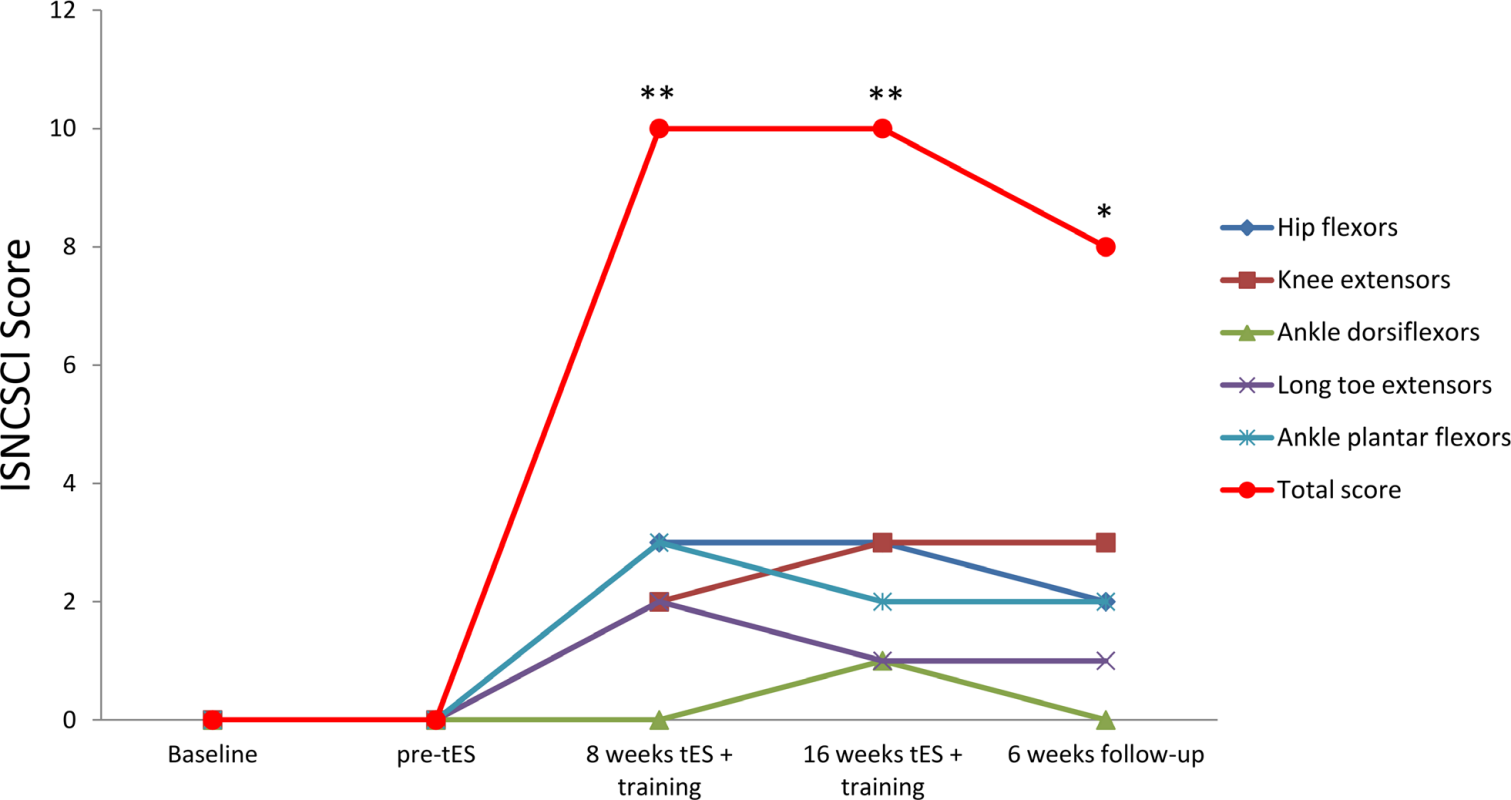
Assessments of International Standards for Neurological Classification of SCI (ISNCSCI) of the left leg at the baseline, pre‐tES, after 8‐ and 16‐week tES with training, and at 6‐week follow up without spinal cord stimulation or training. The total score of the five individual movements (hip flexion, knee extension, ankle dorsiflexion, long toe extension, and ankle plantar flexion) exhibited significant improvements (**P < 0.001; one‐way ANOVA, post hoc Tukey’s multiple comparison test) after stimulation as compared to the baseline. The score remains significantly higher than the baseline (*P < 0.05; one‐way ANOVA, post hoc Tukey’s multiple comparison test) even after stimulation and training had been stopped for 6 weeks (follow up).

Lower extremity motor scores (LEMS), according to the international standards for neurological classification of SCI (ISNCSCI), increased significantly (*P* < 0.001; one‐way repeated measures ANOVA; post hoc Tukey’s multiple comparison test) over the course of the tES and training. The baseline total LEMS of the plegic left leg was 0 but the total LEMS score increased to 10 after 8 weeks of tES and motor training (grade 3 voluntary contractions of the quadriceps and tibialis anterior muscles, which were originally paralyzed) (Fig. 5). The right‐leg total LEMS also increased from 17 to 21. The functional improvements, however, did not further improve after another 8 weeks of tES treatment and training. Cessation of tES and training for 6 weeks resulted in a slight drop of motor scores (LMES from 10 to 8), although it remained significantly higher than the baseline score (*P* < 0.05; one‐way repeated measures ANOVA; post hoc Tukey’s multiple comparison test), suggesting a permanent recovery of neurological structures. Furthermore, the tES treatment improved pin prick sensation (from 73 to 79, ISNCSCI score) of the patient (Fig. S1).

## Discussion

Recent landmark studies have demonstrated that the application of invasive epidural electrical neuromodulation (eES) to spinal cord networks caudal to the spinal lesion dramatically improves motor control in patients with SCI when combined with activity‐based therapies such as massed practice and treadmill training.^3^, ^11^, ^12^ However, activity‐based therapies alone showed no benefit on the functional recovery of these patients.^13^, ^14^ Furthermore, in no case have any of the studies observed in animal or human experiments after complete paralysis to be able to train.^15^ Apparently, a paralyzed nervous system cannot be trained if it cannot be activated. In the present study, using non‐invasive electrical stimulation and motor training, we have demonstrated significant improvements in motor function recovery including volitional control of the plegic limb, independent standing and squatting of a patient with cervical cord injury who had been paralyzed for more than 21 years. In this study, we tested two tES parameters at spinal segments near the target lumbosacral spinal cord region to detect the recruitment of the patient's lower limb motor pools in a resting condition. With rigorous analysis of MEP in the lower limbs, we found that shorter burst duration (100 *µ*sec) caused more homogeneous recruitment of motor pools than longer burst duration (1 msec) used in previous studies. Our patient also preferred shorter to longer tES burst, perhaps, because of having better sensation of the paretic areas during shorter tES bursts. Further research is warranted to better understand the relation between burst duration and the functional connection.

Although recent transcutaneous spinal cord stimulation studies have shown improvement of trunk control during sitting^16^ and self‐assisted standing^17^ in individuals with SCI, this is the first study to show that tES can facilitate volitional movements in the completely paralyzed leg of a SCI patient after just 16 weeks of stimulation and training. This finding is comparable with previous research using invasive epidural electrical stimulation on a motor complete paraplegic patient regaining volitional movements of lower limbs after stimulation and training.^4^ Furthermore, the impacts of tES on our patient’s full leg extension and flexion movements were similar to the findings in previous epidural stimulation studies.^5^, ^6^ The improved leg extension facilitated our patient to control her knees to prevent buckling during standing. Similar to a previous study,^9^ our patient became less dependent on the stimulation to control her legs as the training progressed.

In this study, substantial flexor and extensor movements of both legs occurred even in the absence of spinal stimulation after around 32 treatment sessions. To the best of our knowledge, the only study that matches the chronic effect of stimulation was reported by Gerasimenko et al.,^9^ where it was observed that the magnitude of bilateral rhythmic motions of the lower limbs was similar regardless of the presence or absence of the stimulation. In the present study it was noted that coactivations of different muscle groups were obvious during the transition from sitting to standing and vice versa. However, this is almost an universal phenomenon following spinal cord injury, differing only in the degrees of abnormality of coactivations.^18^, ^19^ Prior research has shown that neuromodulation of the lumbosacral enlargement via tES increases the EMG activity in lower‐extremity muscles in SCI patients during treadmill^20^ and robot‐assisted^21^ locomotion. However, in the current study, tES exhibited little improvement in our patient’s bipedal walking. Furthermore, increasing either burst duration or stimulation intensity did not facilitate walking. A previous study also found that higher levels of stimulation interferes with the robustness of stepping pattern.^9^

After 52 sessions of tES and motor training, our patient regained full volitional movements of her paralyzed leg, as well as independent standing and squatting. Following this treatment, the total lower extremity motor score of her left leg improved significantly from a fully paralyzed condition. This was the most significant recovery since her injury 21 years ago. Cessation of tES and training for 6 weeks did not cause the motor scores to return to the baseline level. The fact that the score was still significantly higher compared to the baseline suggests some level of permanency of recovery possibly through neuroplasticity. There was, however, no noticeable post‐treatment change in the spasticity of the lower limbs as measured by the modified Ashworth Scale (MAS) scores. In particular, MAS scores of the bilateral lower limbs were 1+. Blood pressure and resting heart rate at seated position did not change significantly throughout the study. Other post‐treatment autonomic functions such as bladder and bowel control were similar to those at baseline. Some trunk control might have improved over the course of the study as after tES training the patient was able to sit in more upright position on command, however, we did not quantify such functional recovery. The patient did not experience any pain throughout the treatment period, and there was no significant change in tactile sensation, although improved pin‐prick sensation was reported at the end of the study.

This single case study highlights a possibility of using tES as a promising non‐invasive intervention to restore physical functions of individuals with chronic paralysis following a SCI. In the present case, we reported several novel results, in particular, the level of recovery achieved after 21 years of complete paralysis in one leg and severe paralysis in the other, and the ability regained by the patient to voluntarily generate movements of such a magnitude. A recent epidural electrical stimulation study^12^ has demonstrated significant autonomic and some motor function recoveries without simultaneous physical training in chronic SCI patients. However, no non‐invasive stimulation has yet been investigated on the sole effects of electrical stimulation on functional recovery without physical training.

Despite the promising results, the current study has some limitations. First, the current findings may not be generalized to other patients with SCI. Future research with larger sample sizes is warranted to confirm the positive outcomes. Second, it remains unclear whether the position of the stimulation electrodes may have effects on the treatment results. Future studies should be conducted to identify the optimal electrode locations. Furthermore, the mechanism underlying the recovery remains unclear. Future mechanistic studies should be conducted to understand the functional mechanisms of such recovery, which can help determine the optimal stimulation parameters for the best treatment outcomes for these patients.

## Author Contributions

MA, VRE, and YPZ designed the study. HZ performed the initial assessment and testing. MA, YTL, and AYLW collected the data. MA and YTL compiled the data and generated the figures. All authors interpreted the data and wrote the manuscript.

## Conflicts of Interest

The authors declare no conflict of interest.

## Supporting information


**Figure S1.** ISNCSCI worksheet at the baseline (pre‐treatment) and after 16 weeks of tES and training (post‐treatment).

**Video S1.** Volition movements of the left limb in supine position.

**Video S2.** Volitional leg movements after 10 and 20 sessions of tES treatment at T11 and L1 spinal levels with physical training.

**Video S3.** After just 32 sessions of tES and training, the patient could stand in an upright posture with only minimal supports to her ankles to prevent twisting.
