# Supplementary figures and images for: Reversing 21 years of chronic paralysis via non‐invasive spinal cord neuromodulation: a case study
Source: Ann Clin Transl Neurol. 2020 May 20;7(5):829–38. doi: 10.1002/acn3.51051 (PMC7261759; doi:10.1002/acn3.51051)

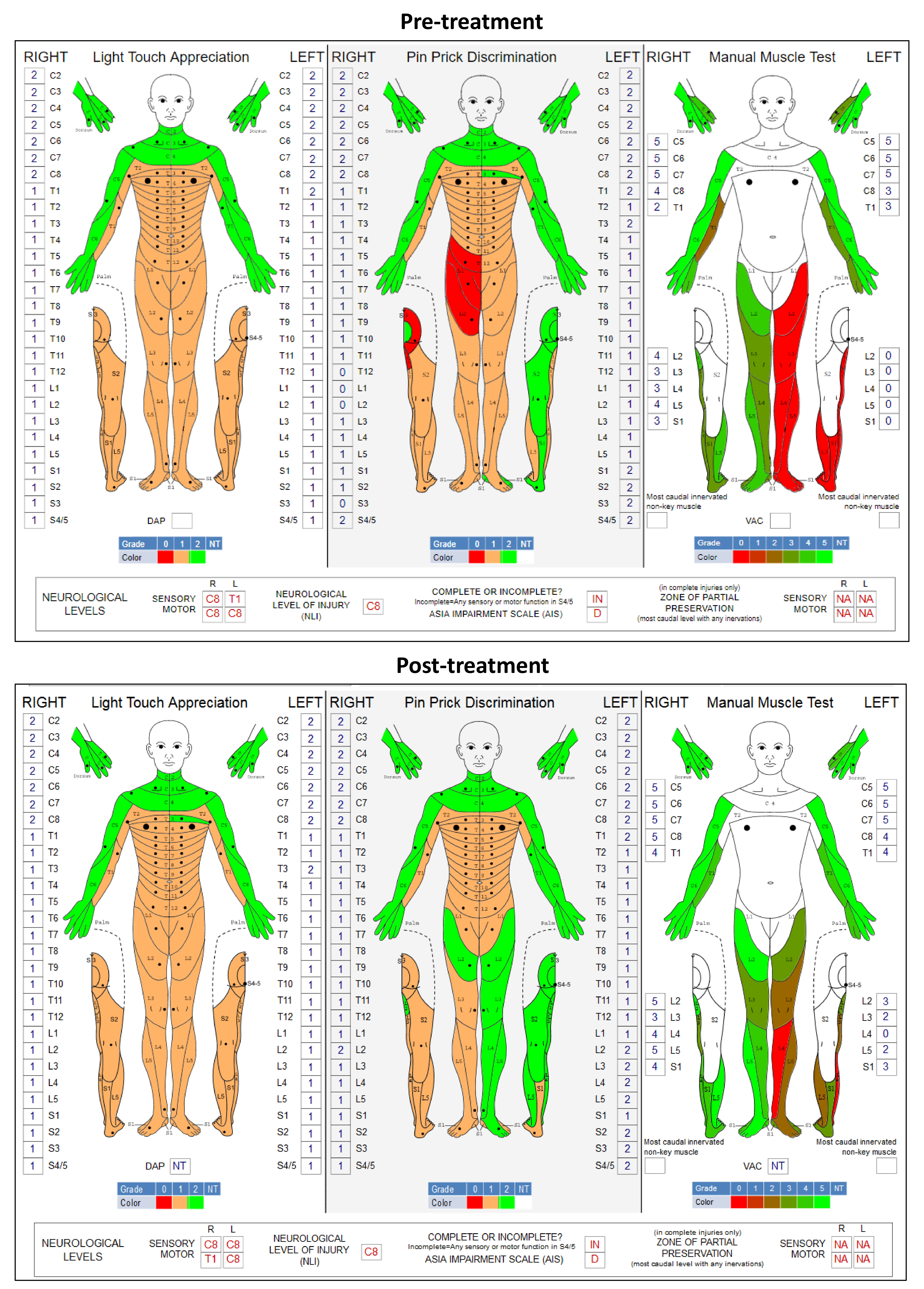

Supplement: Supplementary file 1 — Figure S1. ISNCSCI worksheet at the baseline (pre‐treatment) and after 16 weeks of tES and training (post‐treatment). [file ACN3-7-829-s001.tif]
